# Effects of stubble and mulching on soil erosion by wind in semi-arid China

**DOI:** 10.1038/srep29966

**Published:** 2016-07-18

**Authors:** Peifei Cong, Guanghua Yin, Jian Gu

**Affiliations:** 1 Institute of Applied Ecology, Chinese Academy of Sciences, Shenyang 110016, China; 2 Institute of Geographical Sciences and Natural Resources Research, Chinese Academy of Sciences, Beijing 100101, China; 3 University of Chinese Academy of Sciences, Beijing 100101, China

## Abstract

Soil erosion is a growing challenge for agricultural production in Northern China. To explore the effect of variation in stubble height and mulching biomass on soil erosion caused by wind, we conducted a field experiment using a quadratic rotation combination design. Results showed that the quantity of straw mulch was the dominant factor affecting soil erosion, and stubble height was of secondary importance. The soil water content in stubble and straw mulching treatments was higher than in a control treatment at 0–20 cm soil, and the tendency in the amount of soil water content was opposite to the amount of wind erosion (r = −0.882, n = 10, p < 0.01). The change in soil water content observed in the stubble and mulch treatments at the 15–20 cm depth was higher than the change from 0–5 cm to 5–10 cm. Combined, the influence of a stubble height of 34 cm and mulch quantity of 4260 kg·ha^−1^ lowered the amount of erosion to 0.42 t·ha^−1^, and increased the corn yield to 11900 kg·ha^−1^. We determined that those were the most appropriate levels of stubble height and straw mulch for crop fields in the semi-arid regions of Northern China.

Soil erosion caused by wind is one of the major barriers to sustainable development of agriculture in arid areas. According to China’s second National Remote Sensing Investigation of Water Loss and Soil Erosion, the country’s total area affected by soil and water loss was 3.56 × 10^6^ km^2^, the area of soils affected by water erosion was 1.65 × 10^6^ km^2^ and by wind erosion was 1.91 × 10^6^ km^2^ in the late 1990 s^1^. Wind erosion resulted in serious land degradation and decline in the total area of cultivated land^2^. The factors that affect field wind erosion include soil surface roughness and crop residues.

Hol^3^ developed a model that uses soil roughness to predict wind erosion. Saleh and Fryrear^4^ and Hagen^5^ developed the wind erosion equation to evaluate the effect of wheat crop reducing field wind erosion. Bilbro and Fryrear^6^ developed mathematical relationships between flat residue and standing residue and soil loss ratio, they report the latter to be more effective than the former. Because the standing residue reduced the wind speed. Straw prevents strong winds from eroding the soil due to the protection it provides for the soil particles^7^,^8^. The stubble of straw affected the direct wind exposure. An increase of wheat stubble height from 30 to 61 cm reduced the wind speed by 74% 9.

Leaving crop residues in the field has been demonstrated as an effective method to prevent wind erosion^10^,^11^. Straw mulching can increase soil warming^12^ and affect soil water evaporation^13^,^14^. The stubble height was also an important management decision. Standing crop residues absorb wind energy and lift the zero velocity point above the soil surface^6^. The height and quantity of stems determines the silhouette area, thus ensures the effectiveness of standing crops.

Hagen and Armbrust^15^ proposed a theoretical model that showed a high correlation between plant area and soil wind erosion. The model use two sets of wind tunnel data from Lyles and Allison^16^ and van de Ven *et al*.^8^, which suggest the Plant Area Index (PAI) as an indicator of wind erosion protection by standing plants. Hagen^17^ also proposed that standing residue was more effective than flat residue. In addition, a vertical silhouette area of 5 percent of standing residue per horizontal area unit was adequate to protect soil in low and moderate wind regimes. Mulching quantity and the stubble height were two critical factors influencing crop yields. A feasible plan to balance the two objectives (stubble height and straw mulching quantity) is of key importance for the development of sustainable agriculture.

Our objective was to explore the effect of variation in the stubble height and mulch quantity on soil erosion by wind. We compare the results with different treatment combinations and investigated effects of our interventions on erosion, the physical and chemical properties of soil, and crop yield. We also determined the optimum levels of stubble height and mulch quantity. Our findings contribute to the theoretical basis for measures to control the problem of soil erosion by wind, due to sandstorms, in semi-arid regions of Northern China.

## Experimental Design

The two factors of our experiments were the height of maize stubble (H) and the quantity of maize straw mulch (M). We used five levels of maize stubble height: 0, 5.3, 18.0, 30.7, 36.0 cm, labeled H_1_, H_2_, H_3_, H_4_ and H_5_ respectively. There were also five levels of maize mulch quantity, 0, 658.8, 2250.0, 3841.2 and 4500.0 kg·ha^−1^, labeled M_1_, M_2_, M_3_, M_4_ and M_5_ respectively. We used a two-factor quadratic rotational combination general design processing the character of the predicted variance of treatment combination. It was points with equal distance to the central in the space of test sphere was almost equal. There were nine treatments (H_1_M_1_, H_1_M_3_, H_2_M_2_, H_2_M_4_, H_3_M_1_, H_3_M_3_, H_4_M_2_, H_4_M_4_ and H_5_M_3_), arranged as a randomized block, with conventional rotary tillage used as the control (Table 1). We repeated each treatment three times. The total plot area was 32 m^2^. The crop studied was spring maize (*Zea mays*) variety Qiule no. 2, sown on April 27, 2013, and harvested on October 2 of that year.

## Results

### Effect of stubble and mulch on soil erosion by wind

**Figure 1:**
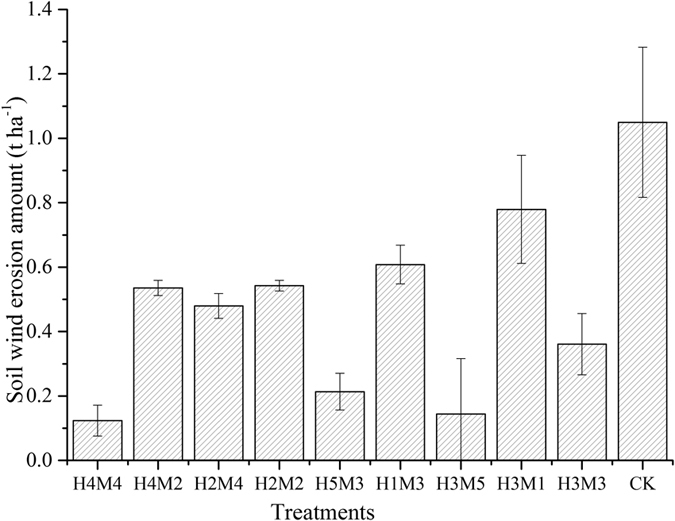
Amount of soil erosion by wind among treatments. The depictend treatments are as follows: H_4_M_4_, height 30.7 mulch quantity 3841.2; H_4_M_2_, height 30.7 mulch quantity 658.8; H_2_M_4_, height 5.3 mulch quantity 3841.2; H_2_M_2_, height 5.3 mulch quantity 658.8; H_5_M_3_, height 36 mulch quantity 2250.0; H_1_M_3_, height 0 mulch quantity 2250.0; H_1_M_3_, height 0 mulch quantity 2250.0; H_3_M_5_, height 18 mulch quantity 4500.0; H_3_M_1_, height 18.0 mulch quantity 0; H_3_M_3_, height 18.0 mulch quantity 2250.0; CK, control. Error bars denote standard errors of means (n = 3).

We calculated the amount of wind erosion for the different treatments (Fig. 1). This showed that wind erosion was greatest with conventional rotary tillage (the control), at 15.74 t·ha^−1^. There was little difference in wind erosion among treatments H_4_M_2_, H_2_M_4_, H_2_M_2_, H_5_M_3_, H_1_M_3_ and H_3_M_3_, which lead to significantly less erosion than the control (p < 0.05), by 42.07–79.67%. The amount of wind erosion in treatments H_4_M_4_ was 1.85 t·ha^−1^, significantly less than other treatments (p < 0.05) and reduced by 88.25% compared to the control. The wind erosion data for different treatments was used to express the relationship between wind erosion (Y), stubble height (X_1_) and mulch quantity (X_2_) as a two-factor quadratic regression model with general rotation:
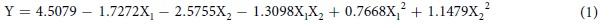


### Factor analysis

Based on model (1), we found no relation between the regression coefficients b_j_, and b_j_ and the regression coefficients of the interaction, or the squared terms Therefore, we used the absolute value of regression coefficients to directly compare the impact of wind erosion. The effect of mulch quantity X_2_ was larger than that of stubble height X_1_. The coefficients of X_1_ and X_2_ were all negative. This indicated that the effect of stubble and mulch can potentially supplement each other. Consequently, if stubble height was increased and mulch quantity reduced accordingly, the amount of wind erosion was invariant.

**Figure 2:**
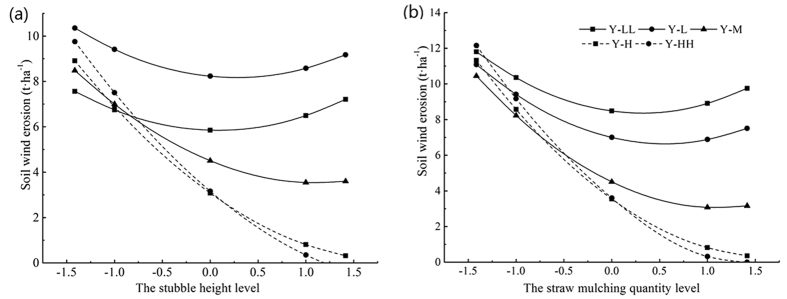
Effect of experimental factors on soil erosion by wind (**a**) Stubble height, (**b**) Mulch quantity. Y-ll indicated the lowest level (both the stubble height and the straw mulching quantity were all at −1.414 level); Y-l indicated the lower level (the two factors were all at −1 level); Y-m indicated the middle level (the two factors were all at 0 level); Y-h indicated the higher level (the two factors were all at +1 level); Y-hh indicated the highest level (the two factors were all at +1.414 level).

Five sub-quadratic regression models were obtained using the dimensionality reduction method, when stubble height or mulch quantity were fixed respectively at levels of −1.414, −1, 0, +1, +1.414. A plot of wind erosion versus stubble height and mulch quantity (Fig. 2) showed that: when X_2_ was fixed at −1.414, −1 and 0 and X_1_ was −0.081, +0.272 and +1.126, respectively, soil erosion was minimal, the corresponding values of Y_H_ were 5.85, 8.17 and 3.54 t·ha^−1^ respectively. Before reaching its lowest point, wind erosion decreased dramatically as stubble height increased. When X_2_ was fixed at +1.414 and +1, the lowest point of wind erosion was non-existent at its lowest point for any level of stubble height. The amount of soil erosion declined as stubble height increased (Fig. 2a). Similarly, if X_1_ was fixed at −1.414, −1 and 0, soil erosion reached its minimum when the values of X_2_ were +0.315, +0.551 and +1.122, the corresponding values of Y_M_ were 8.37, 6.65 and 3.06 t·ha^−1^ respectively. Before reaching the lowest point, soil erosion declined rapidly as stubble height increased. When X_1_ was fixed at +1.414 and +1, the lowest point of wind erosion also did not exit at its point for any level of mulch quantity and soil wind erosion was reduced as mulch quantity increased (Fig. 2b). In conclusion, the code values of stubble height and mulch quantity were (0, +1.414), the intervention measures reduced the amount of erosion caused by wind.

**Figure 3:**
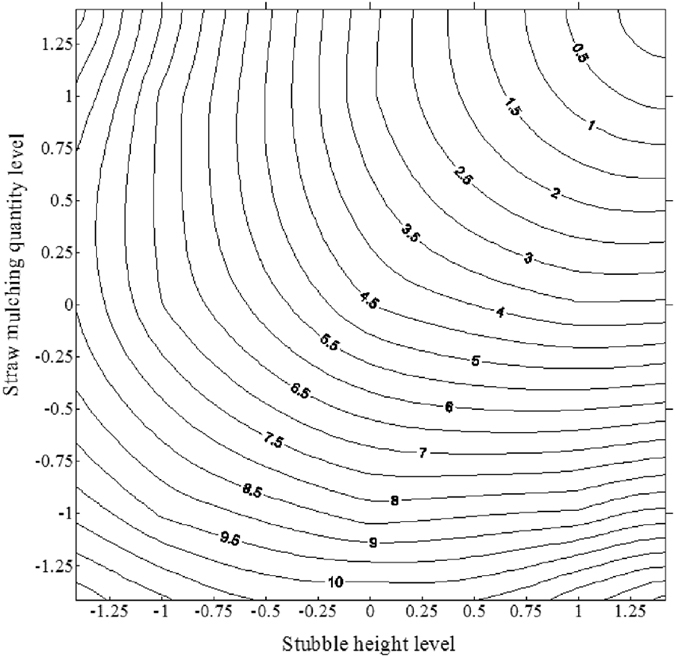
The interaction effect of soil wind erosion between stubble height and mulch quantity.

Based on model (1), we plotted the interaction effect of stubble height and mulch quantity (Fig. 3). When stubble height and mulch quantity were both −1.414, soil erosion reached its maximum of 11.80 t·ha^−1^. The amount of soil erosion decreased as stubble height and mulch quantity increased. If one factor was fixed, soil erosion was reduced when the other factor was increased. When X_1_ = +1.414 and X_2_ = +1.414, erosion reached its minimum of nearly 0. Thus, when stubble and mulch levels changed simultaneously, soil erosion was more sensitive than it was to either single factor.

The minimum amount of wind erosion was obtained using the theory of the extreme value of multiple functions. Based on equation (1), the partial derivatives of both X_1_ and X_2_ were obtained when the equation was equal to zero. Thus, we obtained the value: X_1_ = +1.126 (the equivalent of a stubble height of 33.9 cm), X_2_ = +1.122 (the equivalent of a mulch quantity of 4255.70 kg·ha^−1^), and the amount of erosion by wind was 0.42 t·ha^−1^, which was the minimum value.

### Effects of stubble and mulching on soil water content

Based on analysis of differences in soil water content, the treatments could be divided into four groups for comparison.

The mean soil water content across the growth period of all treatments was highest at 15–20 cm depth (Table 2). The water content trends in treatments H_1_M_3_, H_3_M_3_ and H_3_M_1_ were similar at 0–20 cm depth. The water content variation of the control was larger than that of other treatments at 0–10 cm depth, while in the stubble and mulch treatments, the water content variation at 15–20 cm was more than at 0–10 cm depth.

At seedling stages, when the mulch quantity level was 0, and stubble height levels were −1.414, 0 and +1.414, the soil water content increased with increasing soil depth (Figure S1 and S2). The water content of treatments H_5_M_3_, H_1_M_3_ and H_3_M_3_ was more than the control at 0–20 cm depth. More mulch not only helped to increase the soil water content at 0–5 cm depth, but also reduced the difference in water content among soil layers.

The straw mulching quantity reduced soil water content at different layers when the stubble height level was 0 (Figure S3 and S4). When the stubble height level was −1, and for mulch quantity level of +1 or −1, the soil water content at 0–20 cm and 0–10 cm depth increased significantly (P < 0.05), compared to the control.

At the elongation stage, the soil water content of treatment H_5_M_3_ was higher than other levels at 0–20 cm depth, when mulch quantity was fixed at 0 level (Figure S5 and S6). Water content increased with soil depth as stubble height increased, except for treatment H_3_M_3_ at 10–15 cm depth. The water content was larger at 0–20 cm depth compared to the control and increased with increasing depth (Figure S7 and S8). Except at 10–15 cm depth, when stubble height was 0, the soil water content was greater when mulch quantity level was +1.414 compared to levels of 0 or −1.414. When the stubble height level was −1, soil water content of +1 level mulch treatments was greater than at −1 level.

At the heading stage, the variation of soil water content was more than the jointing stage. When the mulch quantity was fixed at 0 and stubble height was at −1.414, 0 and +1.414, the water content at 10–20 cm depth was more than at 0–10 cm (Figure S9). However, the change in water content of the control at 0–20 cm depth was not significant. The water content at 0–5 cm depth increased significantly by 17.64% (p < 0.05) compared to the control, when both the mulch and stubble levels were +1 (Figure S10). The water content of treatments at 0–5 cm depth increased by 22.43% compared with the control, when stubble level was 0 and mulch level was −1.414, 0 and +1.414 (Figure S11 and S12).

At the maturation stage, the water content of treatments at 0–5 cm depth was larger compared to the control, if the mulch level was fixed at 0 and the stubble heights level were +1.414, 0 and −1.414 (Figure S13 and S14). The soil water content at 0–20 cm depth increased with mulch quantity for treatments with stubble height level was 0 or −1 (Figure S15 and S16).

**Figure 4:**
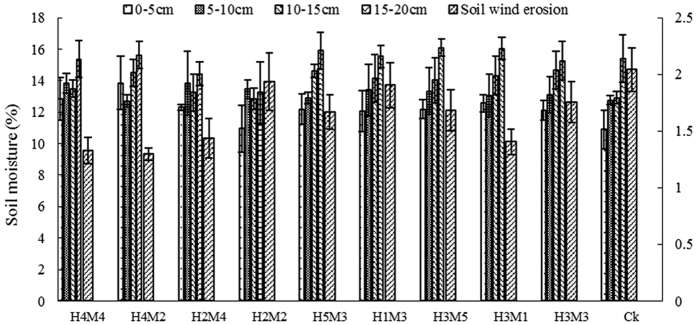
Soil erosion by wind and soil water content at the seedling stage.

At the seeding stage, the variation of wind erosion was obvious among treatments with different soil water contents (Fig. 4). We found a negative correlation between soil water content at 0–5 cm depth and the amount of erosion by wind (r = 0.882, n = 10, p < 0.05).

**Figure 5:**
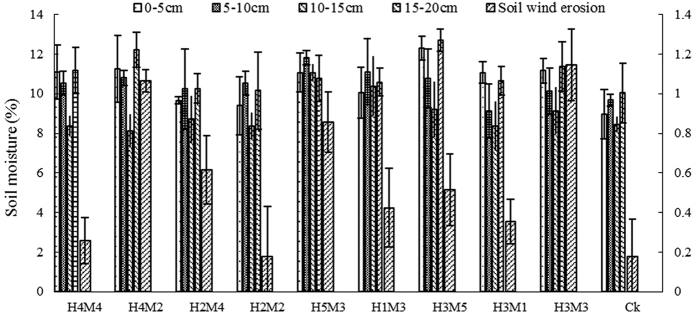
Soil erosion by wind and soil water content at the maturation stage.

At the mature stage, there was a strong negative correlation between soil water content at 0–20 cm and erosion (Fig. 5), probably because straw mulch decayed over time and the effect in covering soil increased, so soil wind erosion reduced. Also, the soil erosion strength had weakened during this period.

The above analyses demonstrated that the amount of wind erosion could decline as the soil water content at 0–20 cm depth increased. It means the soil water content was able to inhibit wind erosion at the soil surface. Our results show that it is possible to reduce soil wind erosion by improving soil water content.

### Effect of stubble and mulch on spring maize yield

**Figure 6:**
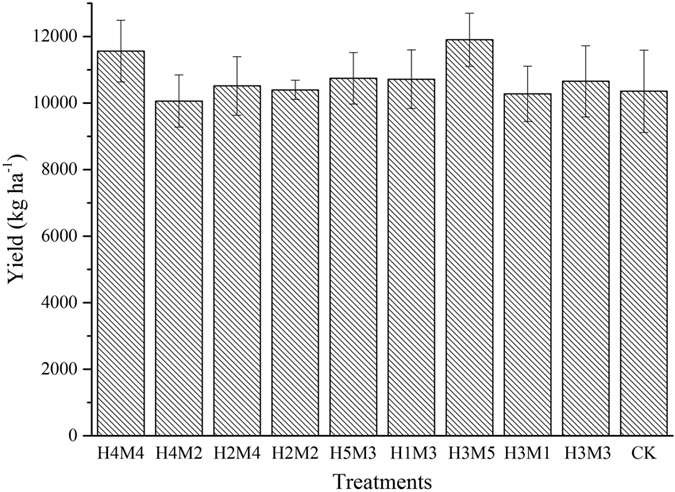
Spring maize yield in different treatments.

The mean maize yield of the control was minimal (Fig. 6), at 10359.0 kg·ha^−1^, but compared to other treatments the difference was not significant (p > 0.05). The maize yield of treatments H_3_M_5_ and H_4_M_4_ (11901.7 and 11560.7 kg·ha^−1^ respectively) were 14.89% and 11.60% higher than that of the control group. The yield of treatments H_3_M_3_ and H_5_M_3_ were 10414.1 and 10742.9 kg·ha^−1^ respectively, increased by 0.53% and 3.71% compared to the control.

**Figure 7:**
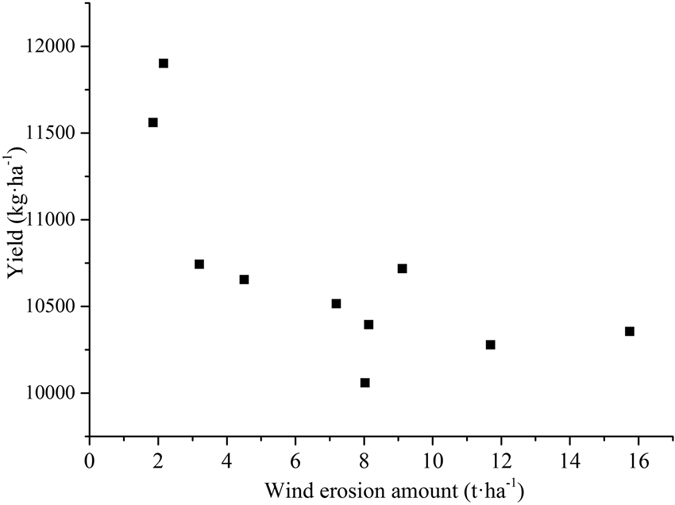
Maize yield and amount of wind erosion.

We found a negative relation between soil erosion and maize yield (Fig. 7). Treatments with lower wind erosion had larger yield, and vice versa. As to the correlation analysis, the result showed that the relationship between the two variables was strong and significant. In the limited range of stubble height and straw mulch quantity, the fitted equation of yield and wind erosion amount was y = 14.111x^2^ − 325.48x + 12077 (r = −0.709, n = 10, P = 0.02). Thus, there was a negative correlation between yield and wind erosion amount. In order to ensure high yields, controlling soil wind erosion is advisable.

Combined with the relationship between soil erosion or yield and the levels of stubble height and mulch quantity, we found that less erosion occurred for a stubble height of 33.9 cm and a mulch quantity of 4255.7 kg·ha^−1^. We therefore determined that those were the optimum intervention levels used in western Liaoning Province, to counteract the effects of frequent sandstorms.

## Discussion

The model (1) equations showed that stubble height and maize mulch quantity determined the effect of the soil erosion caused by wind, and the effect of mulch was greater than that of stubble. This may be due to variable wind speeds among treatments. Crop quantity and height determined the absorbed energy. Nielsen and Aiken^18^ predicted the wind speeds of different stalk populations and cutting heights. Their work showed that when the population was the same, doubling the height would increase the silhouette area index and reduce the wind speeds above the soil surface. These conservation benefits of standing stems results partly from altered wind-speed profiles^19^. The erosive force of wind quantifies the energy available for friction. Different quantity and stem height affect the expected energy of momentum transfer. Increasing the height or population will decrease the energy, so the soil erosion is weakened before the quantity and the height reached limits (Fig. 3).

Increasing stubble height, quantity, or both, reduced evaporation, as well as the soil erosive force (Figure S1–S4), the effect is driven via a slowing of convective vapor exchange and absorbing of radiant energy^20^. The soil water content at 0–5 cm depth increased with mulch quantity, when stubble height was kept constant. If the stubble height was <8.0 cm, little protection was offered. Protection increased with increasing height to 30.9 cm. Synthesizing the data, we tried to seek the most appropriate height and quantity to reach 80% of soil protection and water conservation without reducing a lot of grain yield. The results (Figs 1 and 6) indicated different height and quantity afforded corresponding protection and yield. The relationship between soil protection and grain yield was apparent. In practice, we round these values of stubble height and straw mulching quantity. Because it was easy to operate. Considering measurement error, we also used integers represented the yield data. For example, for a stubble height of 31 cm, and straw mulching quantity of 3840 kg·ha^−1^, erosion reduced by 88.25% and the grain yield increased 14.89% compared to the control. When stubble height was 34 cm and mulch quantity was 4260 kg·ha^−1^, the amount of soil erosion reached a minimum of 0.42 t·ha^−1^ and grain yield reached a maximum of 11900 kg·ha^−1^. We found those to be the most appropriate levels of stubble height and straw mulch for crop fields in Northern China. Stubble height and mulching quantity affected the amount of soil wind erosion according to a change of soil water evaporation. Residue architecture (number and height of standing residue) and the amount of soil covered by loose residue alter the surface microclimate^19^. However, increased stubble height and larger mulch quantity can reduce potential soil water evaporation and convective exchange of water vapor at the soil- atmosphere interface. Moreover, there are optimal level of stubble height and mulch quantity, because lower soil temperatures caused by straw mulch froze the seedlings which negatively influence germination and growth^21^. For example, using a mulch quantity of 4500 kg·ha^−1^, the soil temperature was lower 3–4 °C compared to no mulching treatments. The reduction of the germination rate was significantly. So mulching had an adverse effect to the reduction of wind erosion and increased the yield because of decreased crop density.

The use of measures such as straw mulch and stubble to limit the amount of sand blowing into crop fields are important parts of modern intensive farming in arid areas. We found that the inhibitory action of these interventions was maximized with a suitable combination of the two factors. Therefore, in order to reduce the soil erosion caused by wind and avoid the waste of valuable resources, land managers should determine the appropriate stubble height and straw mulch quantity based on local geographical conditions in their area of interest.

Straw mulching was one main method used to improve the sustainability of agriculture in semi-arid North China. It is important to note that straw may have a detrimental effect on some crops. The varieties of the material used on different kinds of crops varies, the strength of the effect on crops also differs. Therefore more attention, in the future, should be paid to the compounds contained in straw with a focus on plant breeding to cultivate less toxic varieties^22^.

As we studied one growing season in a certain set of local weather conditions, many other factors that we did not consider may impact on wind erosion. Our model, therefore, needs to be studied further, to determine its general applicability. We anticipate that the rate of decomposition should vary with stubble height and mulch quantity, which would in turn influence on soil organic matter and nutrient contents. Further studies should be done to investigate those dynamics.

## Materials and Methods

### Site description

The study area was located in Fuxin Mongolian Autonomous County, in the west of Liaoning Province. The area belongs to the southern margin of the Khorchin sand dunes, a semi-arid region in North-east China (120°E, 41°44′N). Mean rainfall per year is only 480 mm, while the annual evaporation capacity is high, ≤1737 mm. The soil organic C concentration was 1.06 g·kg^−1^. In 2013 the soil had sandy loam texture with 60% sand, 29% silt and 11% clay. The soil water content was low. Crops were often sown in the spring every year, when the strong winds lead to frequent sand storms. The surface of the soil was fully exposed.

### Measurements and analysis

The water content of soil was measured using the drying method. Soil samples was dried in an oven for 8 hours at 105 °C. Soil depth was sampled from 0–20 cm one samples per plot, at 5-cm intervals for a total of 4 sampled soil layers. During the experiment from April-October, during 10 continuous days without rainfall approximately, four rounds of soil sampling were conducted: soil water content was measured at the stages of seeding (April 21), jointing (June 23), tasseling (August 1), and maturity (September 14).

### Amount of soil erosion by wind

Soil erosion caused by wind was measured using the wind erosion circle method^23^. Prior to sowing time in spring, we placed a suitable amount of farmland soil in a wind erosion circle (diameter 25 cm and height 3 cm), after weighing the soil and measuring its water content. The top of the erosion circle was uniform with the soil surface. The bottom of the erosion circle needed to remain in full contact with the circle. It formed a whole between the wind erosion circle and the field’s soil. We used the circle to measure the wet weight of soil and its water content during the autumn harvest.

The formula used to calculate erosion by wind was:
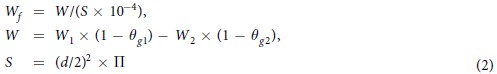
where W_f_ is the amount of soil erosion caused by wind per unit area (kg·ha^−1^), W is the total amount of wind erosion over the entire time period (kg), S is the surface area of the erosion by wind (cm^2^), W_1_ is the weight of soil in the wind erosion circle at the spring sowing stage (kg), W_2_ is the weight of soil in the wind erosion circle at the autumn harvest stage (kg), θ_g1_ is the soil water content in the wind erosion circle at the spring sowing stage (%), θ_g2_ is the soil water content in the wind erosion circle at the autumn harvest stage (%), and d is the diameter of the wind erosion circle (cm).

### Grain yield

At maize maturity, two lateral rows per were discarded in every experimental plot due to the expected edge effect and the remaining middle rows were hand harvested as maize grain yield. The effective area of each experimental plot was appropriately 16 m^2^. We measured the mean fresh ear weight (*G*_1_, kg) of each treatment was measured. Then the mean grain yield, *Y*, via:
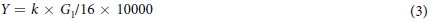
where *k* is the ratio of grain dry weight to fresh ear weight for each treatment. To estimate the values of *k*, ten medium sized ears were sampled from each experimental plot, and we measured the mean fresh ear weight (*G*_2_, kg) and mean fresh grain weight (*G*_3_, kg) for each treatment. The mean moisture contents of fresh grain for each treatment, *A*%, were also determined using a PM-8188 Grain Moisture Tester (Japan). Then *k* was calculated via:
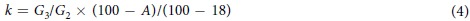


### Statistical analysis

We used the software programs SPSS 16.0, Origin Pro 8.5, Excel 2003 and 8 Surfer to process the data, plot it for exploratory analysis, and perform statistical analysis. The effect of stubble height and mulch quantity on soil erosion was assessed for significance using two factor regression analysis with a model test. The level of P < 0.01 (F = 18.02 >F_0.01_ (5, 7) = 7.46) was very significant. The quadratic regression model was appropriate, indicating that test factors had a significant impact on reducing soil erosion by wind in the study area.

## Additional Information

**How to cite this article**: Cong, P. *et al*. Effects of stubble and mulching on soil erosion by wind in semi-arid China. *Sci. Rep.* **6**, 29966; doi: 10.1038/srep29966 (2016).

## Supplementary Material

Supplementary Information

## Figures and Tables

**Table 1:** Experimental design.

**Treatments**	**Code level**		**Actual value of the factor**	
	**X**_**1**_	**X**_**2**_	**Stubble height (cm)**	**Strew Mulch quantity (kg·ha**^**−1**^)
H_4_M_4_	1	1	30.7	3841.2
H_4_M_2_	1	−1	30.7	658.8
H_2_M_4_	−1	1	5.3	3841.2
H_2_M_2_	−1	−1	5.3	658.8
H_5_M_3_	+1.414	0	36	2250.0
H_1_M_3_	−1.414	0	0	2250.0
H_3_M_5_	0	+1.414	18	4500.0
H_3_M_1_	0	−1.414	18	0
H_3_M_3_	0	0	18	2250.0
CK	−1.414	−1.414	0	0

**Table 2:** Mean soil water content in different treatments.

Soil depth	H_4_M_4_	H_3_M_3_	H_2_M_4_	H_2_M_2_	H_5_M_3_	H_1_M_3_	H_3_M_5_	H_3_M_1_	CK
5 cm	9.81	9.72	9.11	8.61	9.79	9.43	10.21	9.52	9.21
10 cm	9.81	10.26	10.02	9.87	10.30	10.14	10.52	10.25	10.66
15 cm	10.20	10.77	9.83	9.62	11.49	10.57	10.70	10.59	11.01
20 cm	11.36	11.48	10.91	10.50	12.39	11.91	12.20	11.20	11.62
CV	0.0713	0.0710	0.0745	0.0814	0.0868	0.0991	0.0997	0.0672	0.0964

The units of water content are %, CK = control.
