# Supplementary material for: Effects of stubble and mulching on soil erosion by wind in semi-arid China
Source: Sci Rep. 2016 Jul 18;6:29966. doi: 10.1038/srep29966 (PMC4947954; doi:10.1038/srep29966)
Supplement: Supplementary Information [file srep29966-s1.pdf]

Supplementary Information

Effects of stubble and mulching on soil erosion by wind in semi-arid China

Peifei Cong[1,2,3] Guanghua Yin[1*], Jian Gu[1,3]

[1]Institute of Applied Ecology, Chinese Academy of Sciences, Shenyang 110016, China,

[2]Institute of Geographical Sciences and Natural Resources Research, Chinese Academy of Sciences, Beijing 100101, China,

[3]University of Chinese Academy of Sciences, Beijing 100101, China,

* Correspondence and requests for materials should be addressed to Yin, G. H (ygh006@163.com)

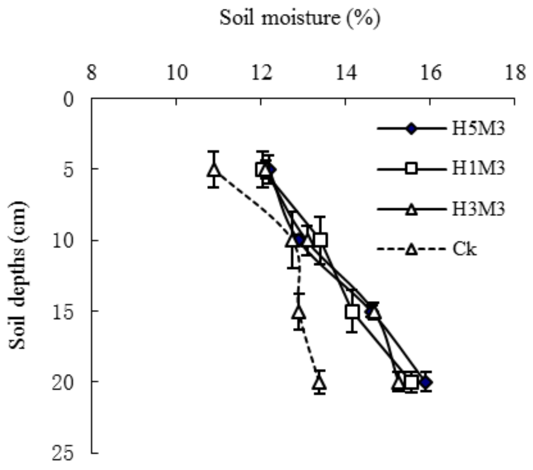

Figure S1 Soil water content of stubble height treatments at the seedling stage (M=0)

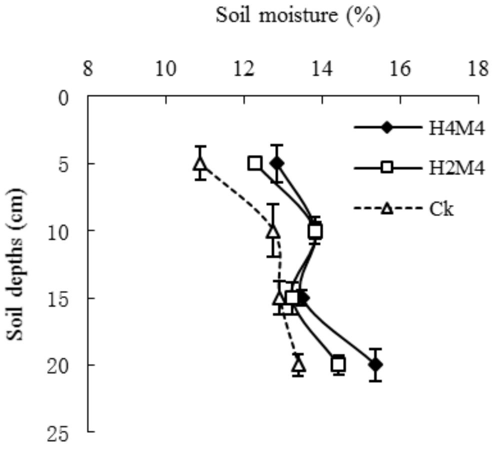

Figure S2 Soil water content of stubble height treatments at the seedling stage (M=+1)

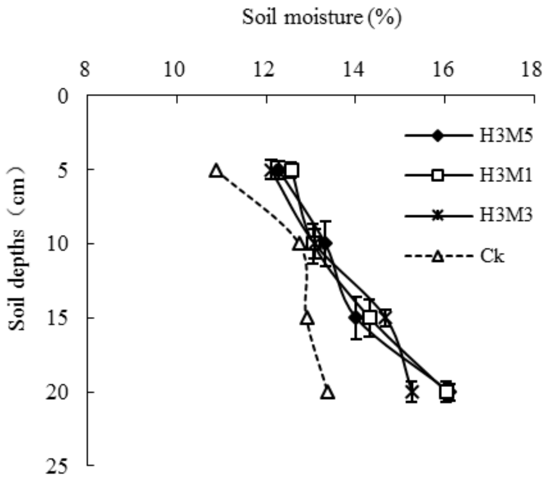

Figure S3 Soil water content of mulching treatments at the seedling stage (H=0)

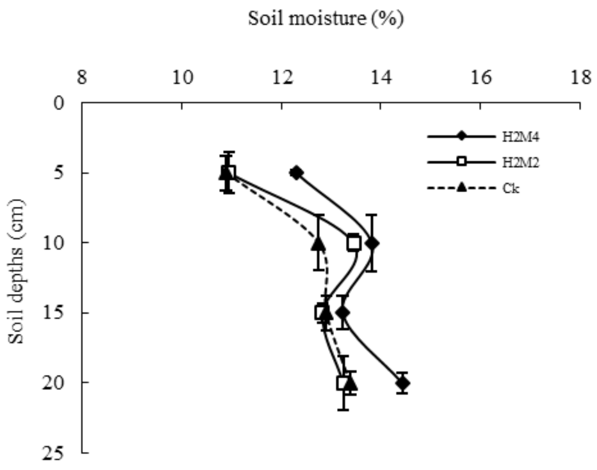

Figure S4 Soil water content of mulching treatments at the seedling stage (H=-1)

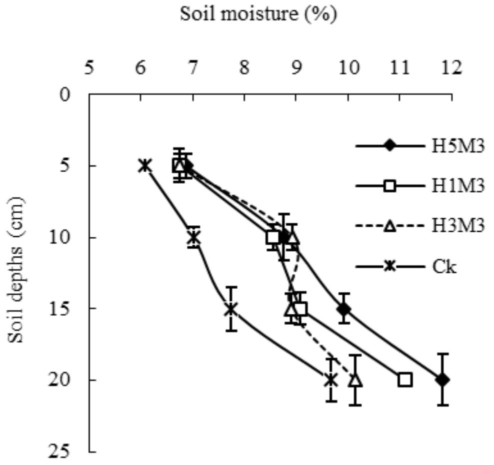

Figure S5 Soil water content of stubble height treatments at the elongation stage (M=0)

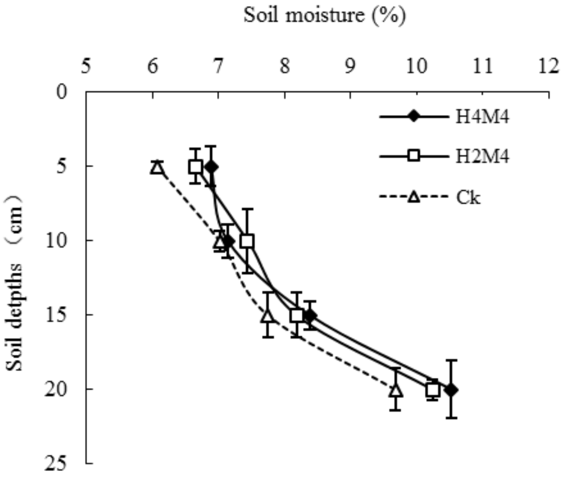

Figure S6 Soil water content of stubble height treatments at the elongation stage (M=+1)

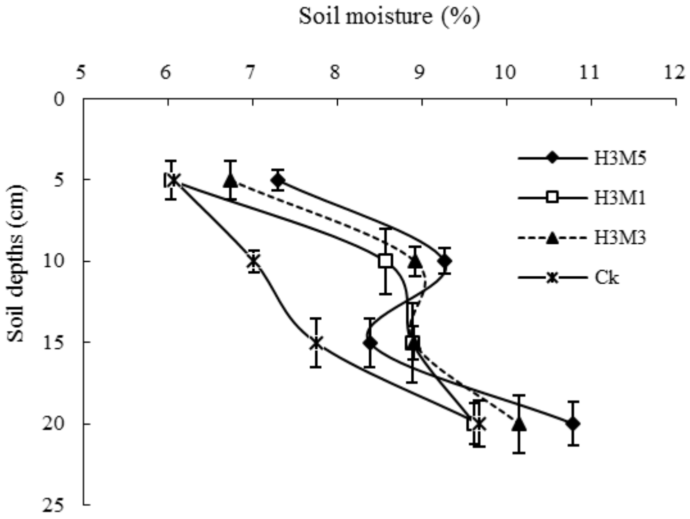

Figure S7 Soil water content of different mulching treatments at the elongation stage (H=0)

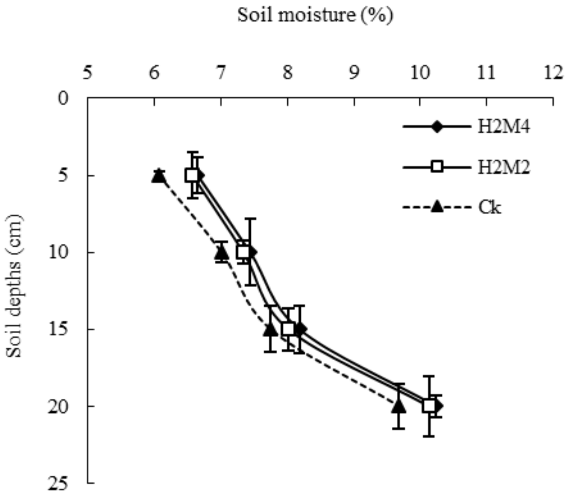

Figure S8 Soil water of mulching treatments at the elongation stage (H=-1)

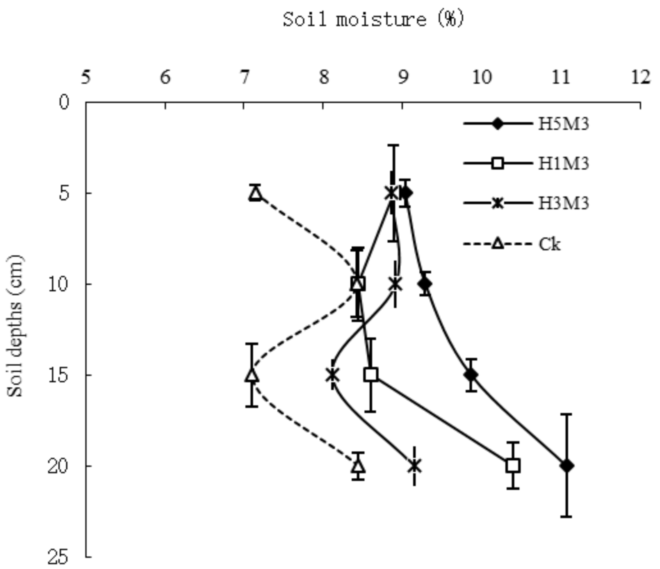

Figure S9 Soil water content of stubble height treatments at the heading stage (M=0)

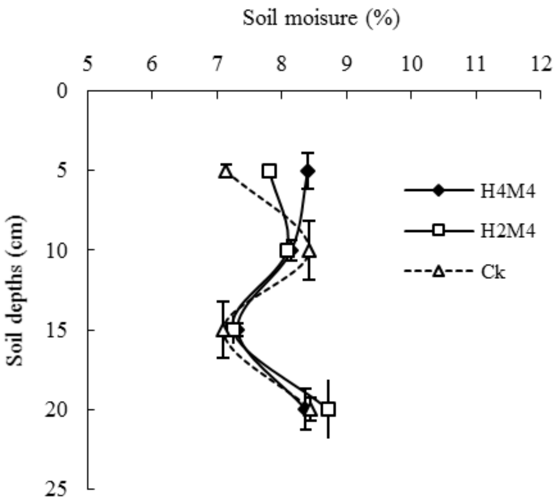

Figure S10 Soil water content of stubble height treatments at the heading stage (M=+1)

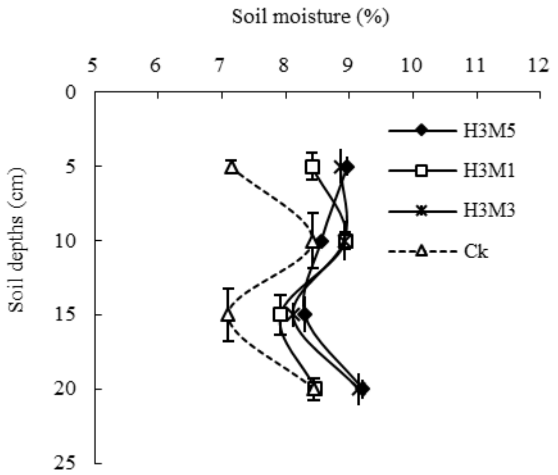

Figure S11 Soil water content of mulching treatments at the heading stage (H=0)

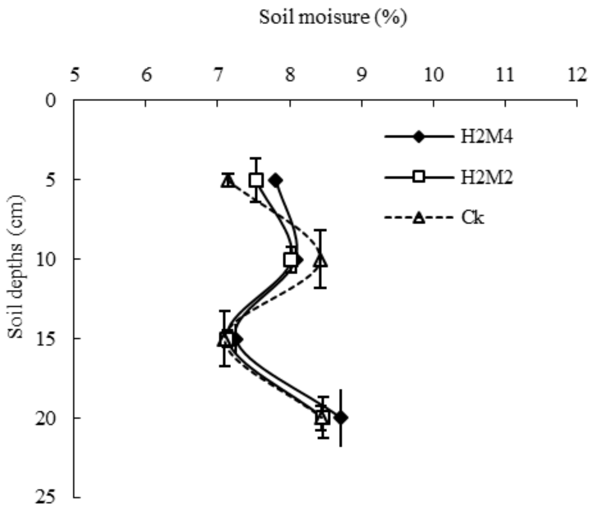

Figure S12 Soil water content of mulching treatments at the heading stage (H=-1)

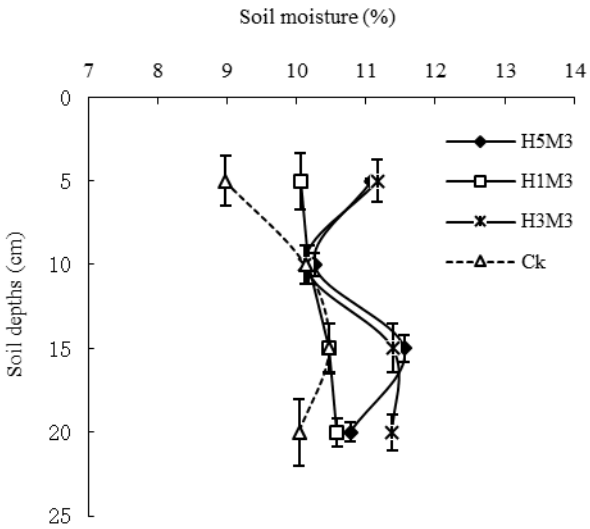

Figure S13 Soil water content of stubble height treatments at the maturation stage (M=0)

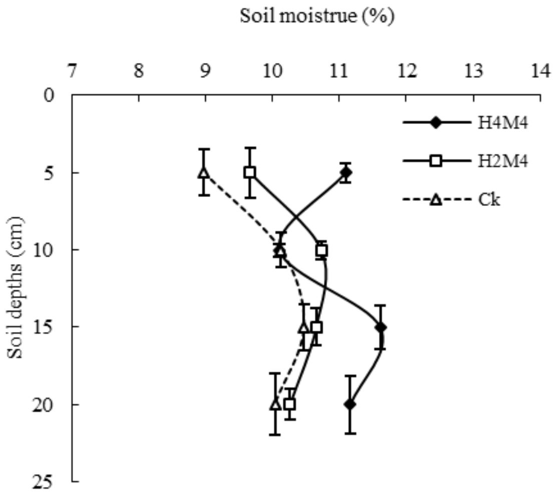

Figure S14 Soil water content of different stubble height treatments at the maturation stage (M=+1)

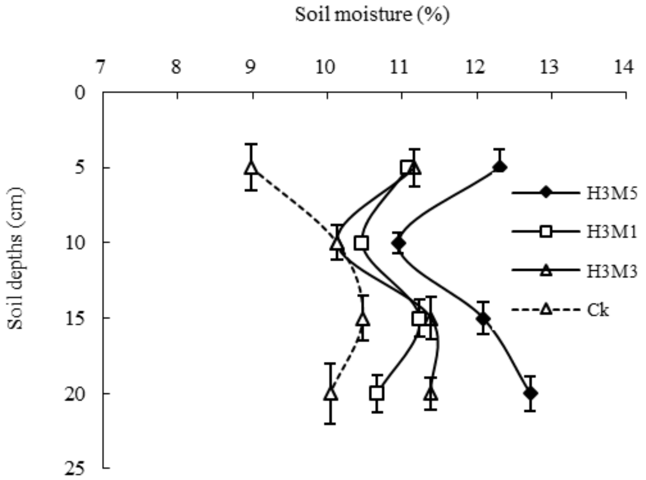

Figure S15 Soil water content of mulching treatments at the maturation stage (H=0)

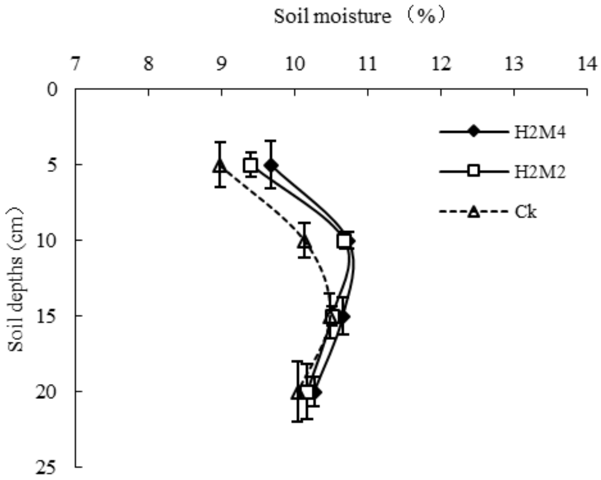

Figure S16 Soil water of mulching treatments at the maturation stage (H=-1)
